# Development of neurodevelopmental disorders: a regulatory mechanism involving bromodomain-containing proteins

**DOI:** 10.1186/1866-1955-5-4

**Published:** 2013-02-20

**Authors:** Junlin Li, Guifang Zhao, Xiaocai Gao

**Affiliations:** 1 Key Laboratory of Resource Biology and Biotechnology in Western China, Ministry of Education, College of Life Science, Northwest University, Xi’an 710069, People’s Republic of China; 2 Institute of Population and Health, College of Life Science, Northwest University, Xi’an 710069, People’s Republic of China; 3 Current address: Taibai Road 229#, Xi’an 710069, People’s Republic of China

**Keywords:** Bromodomain, Chromatin acetylation, Transcriptional regulation, Neurodevelopmental disorder

## Abstract

Neurodevelopmental disorders are classified as diseases that cause abnormal functions of the brain or central nervous system. Children with neurodevelopmental disorders show impaired language and speech abilities, learning and memory damage, and poor motor skills. However, we still know very little about the molecular etiology of these disorders. Recent evidence implicates the bromodomain-containing proteins (BCPs) in the initiation and development of neurodevelopmental disorders. BCPs have a particular domain, the bromodomain (Brd), which was originally identified as specifically binding acetyl-lysine residues at the N-terminus of histone proteins *in vitro* and *in vivo*. Other domains of BCPs are responsible for binding partner proteins to form regulatory complexes. Once these complexes are assembled, BCPs alter chromosomal states and regulate gene expression. Some BCP complexes bind nucleosomes, are involved in basal transcription regulation, and influence the transcription of many genes. However, most BCPs are involved in targeting. For example, some BCPs function as a recruitment platform or scaffold through their Brds-binding targeting sites. Others are recruited to form a complex to bind the targeting sites of their partners. The regulation mediated by these proteins is especially critical during normal and abnormal development. Mutant BCPs or dysfunctional BCP-containing complexes are implicated in the initiation and development of neurodevelopmental disorders. However, the pathogenic molecular mechanisms are not fully understood. In this review, we focus on the roles of regulatory BCPs associated with neurodevelopmental disorders, including mental retardation, Fragile X syndrome (FRX), Williams syndrome (WS), Rett syndrome and Rubinstein-Taybi syndrome (RTS). A better understanding of the molecular pathogenesis, based upon the roles of BCPs, will lead to screening of targets for the treatment of neurodevelopmental disorders.

## Review

Neurodevelopmental disorders are complicated diseases. Studies are currently underway to understand their regulatory mechanisms, to explore better treatments and, ultimately, to prevent their onset.

Chromatin modification traits are associated with the pathogenic characteristics of many neurodevelopmental disorders. Bromodomain-containing proteins (BCPs), which contain a bromodomain (Brd), represent a family of proteins found in many chromatin-associated proteins and in a range of transcription factors. A hydrophobic pocket formed by the ZA loop and BC loop is responsible for acetyl-lysine recognition and specific binding, the accuracy of which could be improved by sequence and acetyl-lysine modification of target sites. Thus, several inhibitors targeting these interactions have been designed for potential clinical application.

BCPs play pivotal roles in transcription regulation. Some BCPs participate in initiation and regulation of basal transcription by binding nucleosomes. However, as targeting proteins, some BCPs can recruit their binding substrates and bind their target through the Brds as the ‘reader’ or a recruitment platform for modular protein complexes. They regulate gene transcription by both activation and repression. Conversely, some BCPs can be recruited by other transcription factors (such as p53), and function as modifying enzymes. Consequently, this affects the transcription of downstream genes of other transcription factors.

Abnormal BCPs or BCP-containing complexes in the nervous system have been shown to cause neurodevelopmental disorders, including neural tube defects (for example, GCN5 or BRD2), mental retardation (for example, BRWD3), Williams syndrome (WS; for example, BAZ1B), Rett syndrome (for example, SWI/SNF), Rett syndrome and Rubinstein-Taybi syndrome (RTS; for example, CBP/p300), and Fragile X syndrome (FRX; for example, CBP/p300).

Although the available inhibitors are not efficient enough to be used clinically, for example, by simply increasing histone acetyltransferase (HAT) activity, recent evidence has provided the starting point to investigate BCP’s role in nervous system development and neurodevelopmental disorders.

### Introduction

Neurodevelopmental disorders are a group of diseases characterized by impairment of the growth and development of the brain or central nervous system. Most neurodevelopmental disorders affect behavior, resulting in economic and mental problems, not only for the patients, but also for their families and society. The most common forms of these disorders include mental retardation, autism, bipolar disorder, schizophrenia, attention-deficit/hyperactivity disorder, and learning and memory impairment [1,2].

Understanding the regulatory mechanisms of these diseases is vital for developing better treatments and for preventing their onset. Neurodevelopmental disorders are thought to be caused by (a combination of) genetic and environmental factors. Epigenetic modification is affected by environmental factors, such as drugs, nutrition, toxicities, and mental stress, and its traits are in line with the pathogenic characteristics of neurodevelopmental disorders. Epigenetic modification of chromatin provides a dynamic platform for regulating the expression of target genes via acetylation, phosphorylation, methylation, ubiquitination, and sumoylation [3]. Among such intricate regulatory mechanisms, the equilibrium between global histone N-terminal deacetylation and acetylation changes in response to environmental stimulation through the functional interplay between HATs and histone deacetylases (HDACs). When the equilibrium is disturbed in the brain or central nervous system, patients develop neurodevelopmental disorders with complicated phenotypes.

In the present review, we focus on the roles of BCPs in the pathogenesis of neurodevelopmental disorders. BCPs represent a family of proteins found in many chromatin-associated proteins or in nearly all known nuclear HATs, and contain particular Brds. The importance of BCPs is underscored by the fact that genetic alterations of these genes and the subtle balance of aberrant acetylation are strongly linked to complicated diseases, such as cancer and neurodevelopmental disorders (Table 1). Here, we focus on the key advances in the current understanding of BCPs in the pathogenesis of neurodevelopmental disorders [4]. Special attention is paid to FRX, WS and RTS.

**Table 1:** BCPs implicated in neurodevelopmental disorders

**Protein**	**Name**	**Alias**	**BRDs**	**Protein functions**	**Neurodevelopmental disorders**	**Reference**
BAZ1A	Bromodomain adjacent to zinc finger domain, 1A	ACF1, WALp1, WCRF180	1	Chromatin remodeling factor	Williams syndrome	[5,6]
BAZ1B	Bromodomain adjacent to zinc finger domain, 1B	WSTF, WBSCR9	1	Chromatin remodeling factor, transcriptional regulator	Williams syndrome	[6,7]
BRD2	Bromodomain-containing protein 2	FSH, RING3	2	Increase transcription of E2F- regulated genes	Juvenile myoclonic epilepsy	[8]
BRD3	Bromodomain-containing protein 3	ORFX, RING3L	2	Transcription factor	Autism spectrum disorder	[9,10]
BRWD3	Bromodomain and WD repeat-containing protein 3	BRODL	2	Transcription factor	Mental retardation	[11,12]
CECR2	Cat eye syndrome critical region 2		1	Chromatin remodeling factor	Anencephaly	[13]
CREBBP	CREB-binding protein	CBP, KAT3A	1	HAT, transcription factors, transcription initiator	Rubinstein-Taybi syndrome	[14]
EP300	E1A-binding protein p300	p300, KAT3B	1	HAT	Rubinstein-Taybi syndrome 2	[15]
GCN5L2	General control of amino acid synthesis 5-like 2	KAT2A, GCN5	1	HAT	Neural tube defects	[16]
SMARCA2	SWI/SNF-related matrix associated anti-dependent regulator of chromatin a2	BRM, SNF2L2	1	Chromatin remodeling factor	Nicolaides-Baraitser syndrome	[17]
SMARCA4	SWI/SNF-related matrix associated actin-dependent regulator of chromatin a4	BRG1, SNF2L4, SNF2LB	1	Chromatin remodeling factor	Mental retardation	[18]
TAF1	TAF1 RNA polymerase II, TATA box-binding protein (TBP)-associated factor	TAF_II_250	2	Transcription initiation	X-linked dystonia-parkinsonism	[19]

BCPs, bromodomain-containing proteins; BRDs, bromodomains.

### Background of BCPs

The Brd, first identified in the *Drosophila* brahma (BRM) protein, is a highly evolutionarily conserved domain. More than 75 human BCPs have been identified in the National Center for Biotechnology Information (NCBI) protein database using Brd sequences as query sequences [20,21]. With their particular domains and special functions, BCPs have been identified in a number of nuclear proteins, such as methyltransferases (for example, Ash1 and MLL) and HATs (for example, GCN5 and PCAF), as constituents of chromatin-remodeling complexes (for example, BAZ1A and BAZ1B) and in transcription factors (TFs) (for example, BRD1-4 and BRWD3). Here, we briefly introduce the structure and functions of BCPs, and their relevance to the pathogenesis of neurodevelopmental disorders.

The spatial structure of Brd results in recognition and binding of target regions. Other critical domains in BCPs, the amino acid sequences and posttranslational modifications of target sites combine to subtly and accurately regulate both the chromatin state and the transcription of target genes.

**Figure 1:**
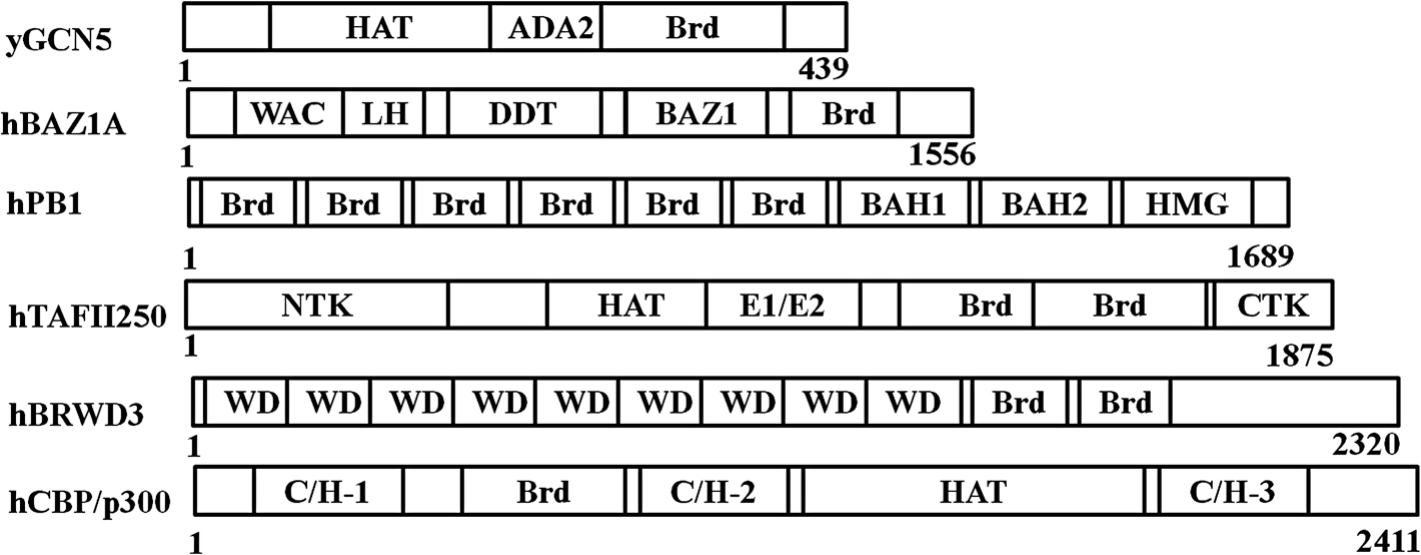
**Bar diagram of representative BCPs and their major associated domains.** ADA2, adaptor 2; BAH, bromo-adjacent homology (1 and 2); BAZ1, bromodomain adjacent to zinc finger domain; BCPs, bromodomain-containing proteins; Brd, bromodomain; C/H, Cys/His rich regions (1, 2 and 3); CTK, C-terminal kinase domain; DDT, DNA binding homeobox and different transcription factors; E1/E2, ubiquitin-activating/conjugating domain; HAT, histone acetyltransferase domain; HMG, high-mobility group domain; LH, leucine-rich helical domain; NTK, N-terminal kinase domain; WAC, WSTF/Acf1/cbp146; WD, tryptophan-aspartic acid dipeptide domain.

Originally, Brd was described as an approximately 60 amino acid domain forming two α-helices. After analyzing the Brds of several BCPs, Jeanmougin *et al.* identified a larger domain of about 110 residues [22]. Based on the resolved structure of Polybromo BRG1-associated factor (PCAF), a typical Brd forms an atypical left-handed, four-helix bundle topology, which includes A, B, Z, and C helixes paired in loops. A hydrophobic pocket formed by a combination of the Z-A loop with the B-C loop is responsible for acetyl-lysine recognition and specific binding [23,24]. Brds may exist at the N- or C- terminus or in the center of proteins, with a repeat number varying from one to six (Figure 1). It is not clear whether the repeated Brds within a protein bind different targets or are a product of natural redundancy. One possible explanation is that the other Brds bind different specific substrates as integration platforms, or allocate the proteins to different nuclear compartments. Self-dimerization of some Brds plays a crucial role in their binding capacity. Using structural data, Nakamura *et al*. determined that Brd1 of BRD2 forms an intact homodimer, in the crystal and also in solution. Brd1 recognizes acetylated H4K12, whereas the dimer interface of Brd1 binds to hypoacetylated H4K8 [25]. Brd2 of BRD2 is monomeric in solution. The affinity between Brd2 of BRD2 and the acetylated H4K12 peptide is as low as 2.9 mM. This implies that BRD2 Brd1 and Brd2 have distinct roles in BRD2’s function [26]. Other BCPs can form dimers, including BRD4, TF_II_D, TAF1, and BRDT. Self-dimerization may help to avoid erasing histone codes during mitosis [27] or might improve modification accuracy [28].

Brds can specifically bind target sites, and the other domains in BCPs contribute to recruit partner proteins or play other critical roles. For example, in p300/CBP, these other domains include three cysteine-histidine (CH) rich domains (CH1, CH2, and CH3), a KIX domain, and an ADA2-homology domain (Figure 1). In particular, both the PHD finger and Brd of p300 are required for interaction between p300 and nucleosomes. P300 binds nucleosomes in an acetylation-dependent manner when the PHD finger is combined with Brd. When both of these domains are isolated, the PHD finger binds to nucleosomes in an acetylation-independent manner, while the isolated Brd cannot bind nucleosomes at all [29,30]. What is the relationship between Brd and PHD in p300? Increasing evidence shows that they form a cassette in BPTF, MLL, and TRIM24 proteins. The MLL PHD domain binds methylated H3K4 and Brd [31,32]. PHD fingers not only recognize an acetyl-lysine residue of histone tails, as does Brd, but also recognize a wide variety of peptides, either modified by methylation, acetylation, or unmodified [32-34].

Complexes containing BCPs subtly and accurately regulate transcription through substrate recognition and by selecting and coordinating between Brds and other domains of BCPs. Brds specifically bind acetylated lysine residues, and the amino acid sequence surrounding the target site is also important for their binding ability [24,35]. Significantly, a recent study showed that the flanking posttranslational modifications on targeting sites, such as phosphorylation and acetylation, are critical for Brds’ specific recognition and binding of substrates [33]. For Brds and other domains of BCPs, recent studies showed their combination improved the accuracy of the regulatory roles of BCPs. In p300, the Brd is important in maintaining the basal activity of HATs and in inducing transcription of the target genes [36]. Ivanov *et al*. uncovered an interaction between the PHD domain and Brd, where the PHD domain of the KAPI corepressor functions as an intramolecular E3 ligase for sumoylation of the adjacent Brd [37]. Based on the spatial reorganization and binding between Brds and their substrates, various inhibitors have been identified as specifically antagonizing the interaction between acetylated histones and Brds. These inhibitors include ischemin [38], novel benzodiazepines, benzotriazepine [39], and I-BET (a derivative of benzodiazepine) [40]. These inhibitory benzodiazepines have anti-inflammatory effects, and have potential benefits for treating specific carcinomas [41-43]. The results from inhibitor studies suggest the important roles of the fine regulation mechanism during normal and abnormal development. More detailed regulatory information, novel targets, and new inhibitors represent promising strategies towards delaying or halting the progression of neurological disorders.

BCPs have multiple functions that rely on particular domains that modify or recruit proteins to form regulatory complexes in response to developmental and environmental cues.

BCPs are implicated in the regulation of cellular events, including the cell cycle, growth, proliferation, differentiation, and apoptosis. Deletion models of different BCPs show a wide range of effects, such as lethality, slow growth, or no phenotypic change [44].

BCPs were initially considered as activators. The transcriptional coactivators CBP/p300, key members of the BCP family, participate in RNA polymerase II-mediated transcription. CBP/p300 possesses HAT activity and transfers an acetyl group from acetyl coenzyme A to a recipient lysine residue. Thus, CBP/p300 loosens dense chromatin, influences global chromatin acetylation and subsequently regulates transcription [45,46]. The binding between p300 and its target gene promoters provides an accessible chromatin structure directly. Indirectly, another key property of CBP/p300 is as a protein bridge, providing a platform for other BCPs and transcriptional regulators [47]. A number of proteins bind to CBP/p300 through the CH1, CH3, and the KIX domains, which are important in mediating protein interactions and in the regulation of cellular events. The interaction between CBP/p300 and E1A is important in cell cycle regulation, involving the control of DNA synthesis and S phase progression, whereas the CBP/p300-p53 interaction is involved in cell apoptosis. The transactivation activity of p53 is improved after acetylation by CBP/p300, which is accompanied by a concomitant increase of the half-life of p53. After DNA damage, p53 might be acetylated by CBP through the Brd binding lysine 382-acetylated p53 peptide. Transcription of cyclin-dependent kinase inhibitor p21 is then activated to induce cell cycle arrest, senescence, or apoptosis. In this process, CBP acts as a HAT and regulates the transcription of p53 downstream genes through p53 binding target sites [48].

Accumulating evidence suggests that the same BCP would function as an activator or a repressor in different situations. For example, BRD7 is a component of chromatin-remodeling complexes, acting as either an activator or an inhibitor. The complex consisting of BRD7 and interferon regulatory factor 2 in the nucleus may activate chromatin transcription *in situ*. In the case of breast cancer with wild-type p53 and deletion of BRD7, the latter has been suggested as a cofactor in the transcriptional regulation of p53 target genes. In the complex constituted by BRD7 and p53, BRD7 affects p53 acetylation and the promoter activity of p53 target genes [49]. In the animal model, when GCN5 is deleted, mice die with increasing apoptosis. The mice survive longer if p53 is also deleted [16]. In addition, BRD7 promotes TCF4-mediated transcription through interaction with β-catenin and TCF4. A model has been proposed whereby BRD7 brings PTP-BL to the Dvl-1/axin/APC/GSK-3β/β-catenin complex, which results in enhanced Wnt signaling. This leads to GSK-3β dephosphorylation and nuclear translocation of β-catenin [50]. The BRD7-containing complex positively influences Wnt signaling; therefore, it is believed to be associated with gene activation. However, there are other types of complexes where BRD7 functions as a repressor. BRD7 can form hSWI-SNF complexes with PRMT5 and PRC2, which leads to inhibition of the expression of suppressor of tumorigenicity 7 (ST7) and retinoblastoma-like protein 2 (RBL2) [51]. In addition, reported substrate affinities range from micromolar to millimolar dissociation constant values [33]. The weak affinities suggest that BCPs possess a dual function (activation and repression). The hypothesis further suggests that BCPs form a recruitment platform or pool for holding different chromatin proteins and function to activate or repress transcription in a temporal-spatial pattern. The duality of BCPs depends on the recruitment of transcription factors and chromatin-remodelers in response to cellular or environmental signaling. Increasing data concerning the structure and function of Brds and BCPs will contribute to understanding the roles of BCPs in normal and abnormal nervous system development.

### Association between BCPs and neurodevelopmental disorders

Abnormal brain development or injury during the fetal stage and during childhood leads to neurodevelopmental and psychiatric disorders. The genetic bases of certain neurodevelopmental disorders have been known for decades. Specific genes have been reported to be associated with neurodevelopmental disorders, but studies on their functional relevance are still ongoing. BCPs are involved in embryonic development and neural malformation [52]. Perhaps the most convincing evidence of the importance of BCPs and their complexes is as follows: even if the DNA sequences of all genes that directly control nervous system development and function are normal, abnormal modification by mutant or dysfunctional BCPs would result in neurodevelopmental disorders (Table 1). Thus, we pay particular attention to BCPs’ roles as TFs, HATs and chromatin-remodeling modulators in neurodevelopmental disorders.

Among 20 members of the BCP family, BRD2 and BRWD3 are related to neuronal development as TFs.

During the formation of the nervous system, a transcriptional program precisely determines the number and types of normal neurons. Precise spatiotemporal regulation controls neuron proliferation, differentiation and apoptosis. Although we know that a regulatory network comprising many TFs is required for neuronal development, we have only scratched the surface in our understanding of this process. Neurodevelopmental disorders show high genetic heterogeneity; therefore, it is difficult to screen and obtain all related genes that directly influence neural and mental development. Increasing evidence implies that BCPs are involved in neural development. More than 20 members of the BCP family have been identified as TFs. Here, we use the roles of BRD2 and BRWD3 in neurodevelopmental disorders as examples (Table 1).

BRD2 (also called RING3 and Fsrg1) is an important transcriptional modulator that is expressed in brain vesicles, the neural tube, the spinal cord and dorsal root ganglia. At the cellular level, BRD2 is localized to the nucleus in proliferating cells and to the cytoplasm in differentiating neuronal precursors [53]. *BRD2*^−/−^ mice deviate from normal developmental programs at embryonic day 9.0 (E9.0), and die at E11.5, with a smaller embryonic size and neural tube defects (NTDs) [54,55] (Table 1). The NTDs appear as exencephaly of the hindbrain. The causal mechanism between BRD2 and NTDs remains poorly understood. BRD2 is implicated in the regulation of cellular events, including growth, proliferation, differentiation, and apoptosis. Several associated proteins were identified as forming a BRD2 complex using Brd2 rabbit polyclonal immune-affinity chromatography. Among them, BRD2 combines with E2F to regulate cell cycle by binding to the *cyclin A* promoter [56]. In addition, BRD2 is the constituent of TF_II_D and SWI/SNF complexes. The available evidence suggests that BRD2 plays an essential role as an integrator of transcription and chromatin structure during mammalian embryogenesis and neurogenesis.

BRWD3 contains two Brds and nine WD repeats. The WD repeat domains are responsible for interaction with other proteins to regulate cellular events, such as cell cycle, signaling transduction, and apoptosis. Truncation mutants of *BRWD3* were identified in the etiology of X-linked mental retardation during systematic screening of the X-chromosome coding sequences in 250 families (Online Mendelian Inheritance in Man (OMIM) ID: 300659) (Table 1). The affected males showed symptoms of prominent forehead, long face, large cupped ears and mild-to-moderate intellectual disability [11]. Recently, we demonstrated that the SNP loci rs7049509 and rs12689192 near the second Brd domain were linked with mental retardation [9] and two new mutations in Brds and WD40 domains, which might be the causes of mental retardation related to *BRWD3* (unpublished data). Research into the causative mechanism is underway. BRWD3 is assumed to be involved in modulation of the JAK/STAT signaling pathway [57]. Notably, the mutant phenotype is related with a frame-shift, and the concomitant loss of a key Brd. Thus, BRWD3 might be associated with learning and memory.

HATs play important roles in regulating gene expression during brain development and memory formation. Aberrant BCPs, as HATs, contribute to neurodevelopmental disorders.

GCN5 is a transcriptional co-activator with HAT activity [58]. Mutant GCN5 is associated with NTDs. The structures of GCN5 and its histone substrates are conserved throughout eukaryotes. GCN5 is indispensable for amino acid metabolism, as a component of the ADA (Adapter) and SPT-ADA-GCN5 acetyltransferase (SAGA) transcription complexes [59]. These complexes regulate transcription processes rather than initiation [16]. The structure of the complex revealed that the single Brd of GCN5 would preferentially bind acetylated H4K16. In yeast, GCN5 acetylates other nearby lysine sites [60]. GCN5 participates in neural and mental development. After being treated with a fear-conditioning stimulus, murine GCN5/KAT2A and acetylation levels were upregulated within one hour. Deletion of GCN5 leads to early embryonic lethality with cranial NTDs. Conditional knockout of GCN5 showed that the normal expression level of GCN5 is critical for neural tube closure in mice, suggesting that mutations of *Gcn5* may be associated with increasing risk of NTDs in humans [61] (Table 1). GCN5 acetylates p53 *in vitro* and *in vivo*, and double mutants of *Gcn5* and *p53* mouse embryos survive longer than *Gcn5* null mice. Deletion of *p53* cannot rescue the embryonic lethality caused by the mutant of *Gcn5*. When the HAT activity of GCN5 is abnormal, cranial neural tube closure in mice is defective [16]. These results reveal that GCN5 is required for survival, and the HAT activity of GCN5 is critical for proper neural tube closure. The exact mechanism remains unclear.

Although NTDs are the second commonest disorders among birth defects in humans, their molecular regulation mechanisms remain poorly understood. Available evidence suggests that acetylation is involved in the likely mechanism of the etiology of NTDs. The process of neural tube closure, including shaping, elevating, bending, and sealing, is finely regulated. In addition to the role of GCN5, neural tube closure is related to acetylation-associated proteins such as CBP, p300, and Hat1 [62]. Knockouts of p300 and CBP show similar phenotypes to GCN5, such as embryonic lethality and NTDs. Consistent with this phenotype, mutations in CBP (that is, chromosomal translocations, microdeletions, and point mutations) are associated with the congenital developmental disorder, RTS.

As mentioned previously, CBP/p300 is another important HAT in the BCP family. It is becoming clear that mutations in CBP/p300 cause 55% of RTS (OMIM ID: 180849), which is characterized by mild to severe mental retardation, craniofacial defects, short stature, skeletal abnormalities, broad big toes, and broad thumbs [14,15,63] (Table 1). This syndrome is relatively common, accounting for 1 in 300 patients with mental retardation. Haploinsufficiency of CBP probably contributes to RTS in humans. Mutations of both the PHD finger and Brds have been identified in RTS patients. In mice, heterozygous deletion or truncation of CBP produced a phenotype resembling RTS [15]. Mutations of CBP (Y1175C, E1278K and R1379P) are associated with RTS, again suggesting the importance of HAT activity [64]. In addition, abnormal CBP/p300 can be found in progressive neurodegenerative diseases, such as FRX, myotonic dystrophy, and X-linked spinal and bulbar muscular atrophy [65]. FRX (OMIM ID: 300624) patients usually suffer from learning disabilities, macroorchidism, seizures, anxiety, mood disturbance, and attention problems. In some cases, individuals display autistic symptoms, including poor eye contact, shyness, self-talk, hand flapping, and hand biting. In line with their similar physical symptoms to mental retardation, the affected males present with more typical physical features, such as prominent ears, macrocephaly, a long face, and a high arched palate [66].

CBP and p300 are also implicated in memory formation. P300 of mice is crucial for long-term memory and learning in a histone acetylation-dependent manner. Long-term memory is impaired in CBP knockdown mice, whereas short-term memory is not affected [67]. If the HAT activity of CBP is abolished, long-term memory is impaired, but short-term memory is not affected. The learning ability of mice could be improved after treatment with HDAC inhibitors of the CREB1/CBP complex. Interestingly, long-term memory can also be rescued with HDACs inhibitors [68,69]. Such studies have illustrated the critical roles of HAT activity of BCPs for long-term memory and synaptic plasticity. Therefore, members of the BCP family with HAT activity regulate transcription by acting as a recruiting platform and through their HAT function. Thus, their mutations may influence transcriptional regulation in two ways: by disrupting the recruiting process and by preventing chromatin remodeling. Together with the properties discussed earlier, at the cellular level, the events leading to the processes of normal or abnormal neurodevelopment, such as cell proliferation, differentiation, survival and polarity may be influenced by acetylation/deacetylation homeostasis events mediated by these special BCPs. At a mechanistic level, intensive research on members of complexes and downstream genes will be helpful in providing clues to the underlying causes of neurodevelopmental disorders.

BCPs also function beyond their main role as transcriptional modulators and enzymes. Some BCPs are associated with neurodevelopmental disorders as chromatin-remodeling modulators.

As chromatin-remodeling modulators, some BCPs affect aspects of chromatin remodeling, in addition to transcriptional regulation, such as increasing the efficiency of chromatin assembly and modulation, DNA replication, DNA repair/recombination, and chromosomal alteration. The function of chromosome remodeling appears to be different from that of transcription regulation [70]. The SWI/SNF complex was first identified as a chromatin-remodeling modulator that remodels and increases access of transcription factors to nucleosomes [71,72]. Abnormal compositions of the SWI/SNF complex are involved in α-thalassemia mental retardation, with patients displaying common characteristics, such as severe cognitive delay, α-thalassemia, facial dysmorphism, microcephaly, skeletal and genital abnormalities, and severe neonatal hypotonia [73,74]. The null and dominant-negative gene mutations exhibit defects of the peripheral nervous system in adults, homeotic transformations, and decreased viability [75].

WSTF including the nucleosome assembly complex (WINAC) is another SWI/SNF-type complex that has ATP-dependent chromatin-remodeling activity. It interacts with the vitamin D receptor (VDR) through the Williams syndrome transcription factor (WSTF, also named bromodomain adjacent zinc finger, BAZ1B). WINAC is a key complex for repressing and activating transcription [76], and is required to enable DNA replication through highly condensed regions of chromatin [77]. The BAZ1B protein contains a Brd, a PHD-type zinc finger motif, a WAKZ motif, and a leucine-rich helical domain (Figure 1). BAZ1B is ubiquitously expressed in both adult and fetal tissues, such as limb buds, tail and brain from around E11.5 in the mouse embryo [7]. It is also expressed strongly in the cranial neural crest-derived mesenchyme, which drives facial morphogenesis. Phosphorylation of BAZ1B in a MAPK-dependent manner is important to maintain the WINAC complex assembly [76]. BAZ1B has been previously identified as contributing to WS [78] (Table 1). Total deletion was detected in 50 of 50 WS individuals using fluorescence *in situ* hybridization analysis. WS (OMIM ID: 194050) is a microdeletion or contiguous gene deletion syndrome characterized by hemizygous deletion of 1.5 to 1.8 Mb on chromosome 7q11.23 [78]. The frequency of WS is estimated to be 1 in 10,000. Subjects with WS show typical craniofacial dysmorphology (a small upturned nose with a flat nasal bridge, mandibular hypoplasia, malocclusion, bi-temporal narrowing and prominent forehead), supravalvular aortic stenosis, multiple peripheral pulmonary arterial stenosis, statural deficiency, infantile hypocalcemia and a distinct cognitive profile with mild mental retardation [79,80]. The patients suffer from specific cognitive deficits, including poor visual-motor integration and attention deficit. In terms of molecular pathogenesis, mutations of BAZ1B and dysfunction of WINAC contribute to WS [5]. The phosphorylation of Ser-158 in the WSTF/Acf1/cbpq46 (WAC) domain is essential for maintaining the association between BAZ1B and core BAF complex components, thereby maintaining the ATPase activity of WINAC. As a sensor, BAZ1B can turn on its chromatin-remodeling activity in response to intracellular signaling [81]. DNA sequence mutations or abnormal modification would disrupt the regulatory complex. These studies might support the view that a chromatin remodeler, such as WINAC, plays a key role in the development of nervous systems and the pathophysiology of WS (Table 1).

Deregulation of methylation and BCPs may alter complex networks of gene expression and brain function, contributing to neurodevelopmental disorders.

Brain development is a complicated process, involving neuron proliferation, differentiation, migration, communication, and apoptosis. Genetic deficits and negative environmental exposures may lead to abnormal neurological development. The characteristics of neurodevelopmental disorders strongly coincide with the traits of dysfunctional epigenetic modification at the chromatin level, alone or in combination. Here, we use Rett syndrome (OMIM ID: 312750) as an example to elucidate the contribution of aberrant methylation and dysfunction of BCPs to neurodevelopmental disorders.

Rett syndrome, a progressive childhood neurodevelopmental disorder, is characterized by stereotypies and mental retardation, and autistic behavior in females. The affected individuals show neurodevelopmental defects, such as common hand movements, aberrant gait, and seizures [82]. Rett syndrome occurs in 1 in 10,000 to 15,000 births and is caused by mutations in the methyl CpG binding protein 2 (MeCP2) gene [83]. Most patients have heterozygous mutations in MeCP2. If MeCP2 is heterozygous in mice, the female also exhibits behavioral symptoms. If totally deleted, the mice show severe neurological symptoms [84]. Although MeCP2 was discovered about 20 years ago, we still have limited information about its molecular function. On the one hand, MeCP2 selectively binds CpG dinucleotides in the mammalian genome, mediating transcriptional repression through interaction with a chromatin-remodeling complex. The SWI/SNF complex can be recruited by MeCP2 to heterochromatic foci in living mouse cells in a DNA methylation-dependent manner. The interaction between ATRX and MeCP2 may control expression of MeCP2-binding genes. When the MeCP2-ATRX interaction is disrupted, pathological changes could be identified in a number of X-linked mental retardations, including Rett syndrome [85]. In contrast, if MeCP2 recruits CREB, it acts as a transcriptional activator. The exact mechanism of MeCP2 action remains unclear as it acts as a repressor or an activator, depending on its interacting protein partners. MeCP2 regulates the brain-derived neurotrophic factor gene (*Bdnf*) in resting neurons. Overexpression of *Bdnf* can rescue a subset of RTS-like phenotypes [86]. In summary, increasing data suggest that MeCP2 acts as a transcriptional modulator, repressing genes by binding to methylated CpG DNA or activating genes through chromatin reorganization. Further research is required to understand the molecular pathogenesis of neurodevelopmental disorders based upon the network created by genetic determination and epigenetic modification.

## Conclusions

The growing number of identified BCPs and the links between some of these members and neurodevelopmental disorders underscore the importance of this class of proteins. BCPs, which are ubiquitous and evolutionarily conserved, play pivotal roles in chromatin modifications, remodeling, and transcriptional regulation. Remarkably, all HATs contain Brds, but not all BCPs are HATs. Some of them are now considered as modulators of chromatin remodeling (such as BAZ1A and CECR2), while some are transcriptional regulators (such as BRD2 and BRWD3) (Table 1). Significantly, some BCP complexes are implicated in basal transcriptional regulation through their Brds or the domains of their partners, influencing the expressions of a range of genes. However, many BCPs regulate transcription through Brds or the particular domains of other TFs that bind to the promoters of target genes (Figure 1). BCPs are critical for cellular events; therefore, complicated phenotypes inevitably result from abnormal genotypes of BCPs or from their dysfunctional complexes. Neurodevelopmental disorders associated with BCPs are not limited to those discussed in this review, for example, bipolar affective disorder and schizophrenia [87]. Further studies addressing the continued functional analyses of each BCP family member are required to better assess the physiological roles of these proteins.

In general, BCPs bind to target sites through their Brds, and they regulate transcription together with their binding partners. BCPs not only ‘read’ the histone code [88], but also dynamically alter chromatin status [89]. HATs (containing some BCPs) write markers on nucleosomes, and HDACs erase them from nucleosomes, and the Brds of some BCPs read the markers [90]. Alternatively, some BCPs regulate gene transcription through the binding of their partners to target sites. In the process of hormonal and nutrient regulation, acetyltransferase complexes containing GCN5 repress glucose metabolism via GCN5 acetylation of PGC-1α [91]. The evidence presented in this review does not represent special cases; there are many other members of the BCP family that may adopt this model as modifying enzymes. Many relevant mechanisms remain to be investigated.

Furthermore, histone acetylation can improve the memory and learning ability through direct control of epigenetic suppression of gene expression [92]. This expectation presents an interesting challenge for future research concerning the wide variety of BCPs. Although they are unlikely to restore patients’ intelligence, the HDAC inhibitors SAHA and trichostatin A exhibit potential rescue deficits in long-term emotional memory and recognition memory in animal models of RTS. Thus, it may be possible to enhance quality of life by improving social behavior and to ameliorate the cognitive and motor deficits in neurodegenerative disorders (that is, Huntington’s, Parkinson’s, and Alzheimer’s diseases). Although the current inhibitors are not efficient enough for clinical use because they simply increase HAT activity, emerging advances highlight the potential of applying BCPs and chromosome acetylation in treating neurodevelopmental disorders.

## Abbreviations

Ash1: Absent, small, or homeotic disc1; ATAD: ATPase family AAA domain-containing protein; BAZ1A: Bromodomain adjacent zinc finger, 1A; BAZ1B: Bromodomain adjacent zinc finger, 1B; BCPs: Bromodomain-containing proteins; Brd1: The first bromodomain protein gene; Brd2: The second Brd protein gene; BRM: Brahma; BET: Bromodomain and the extra terminal domain; BPTF: BRD and PHD finger-containing transcription factor; Brd: Bromodomain; CBP: CREB-binding protein; DRG: Dorsal root ganglia; FRX: Fragile X syndrome; HAT domain: Histone acetyltransferase domain; HDACs: Histone deacetylases; LAP finger: Leukemia-associated-protein finger; MeCP2: Methyl CpG binding protein 2; MLL: Mixed-lineage leukemia; NTDs: Neural tube defects; OMIM: Online Mendelian Inheritance in Man; PBAF: Polybromo BRG1-associated factor; PCAF: P300/CREB-binding protein-associated factor; PDE-4: Phospodiesterase-4; P-TEFb: Positive transcription elongation factor b; RTS: Rubinstein-Taybi syndrome; SAGA: Spt-Ada-Gcn5 acetyltransferase; SAHA: Suberoylanilide hydroxamic acid; SNP: Single nucleotide polymorphism; TAF1: TATA-binding protein-associated factor-1; TAZ: Transcription adaptor putative zinc finger; TFs: Transcription factors; Tg: Transgenic; TPA: Tetradecanoyl phorbol acetate; VDR: Vitamin D receptor; VPA: Valproic acid; WD: Tryptophan-aspartate; WS: Williams syndrome; TFIID: transcription initiation factor.

## Competing interests

The authors declare that they have no competing interests.

## Authors’ contributions

JL, GZ and XG made substantial contributions to design of this study, and JL drafted and manuscript, and GZ and XG revised the manuscript. All authors contributed to writing the manuscript. All authors read and approved the final manuscript.

## Authors’ information

JL focuses on developmental biology and pathogenesis of complicated diseases. Recently, his laboratory has identified SNP loci that are positively associated with mental retardation in the Qinba mountain region, northwest China. Both loci rs7049509 and rs12689192 are near the second Brd domain in the BRWD3 gene. After investigating the functional domains and phosphorylation sites of BRWD3, the group is currently studying the molecular mechanism of neurodevelopment based upon BRWD3. GZ is a full professor, the director of Key Laboratory of Resource Biology and Biotechnology in western China. XG is a full professor, and the vice-director of the Institute of Population and Health. Since its inception in 1995, the Institute of Population and Health has been devoted to the study of neurodevelopmental disorders, such as mental retardation and Fragile X syndrome. The authors’ research teams have carried out investigations on the pathogenesis of neurodevelopmental disorders in the Qinba mountain area, where the incidence is about 3%. In addition to environmental factors, genetic deficits are critical. Susceptible loci and several genes have been identified using gene scanning methods.
